# Factors affecting the initiation and continuation of maternal health service utilization among women who delivered in the past one year in Enemay district, East Gojjam, Ethiopia

**DOI:** 10.1186/s13690-021-00689-y

**Published:** 2021-09-28

**Authors:** Anguach Shitie, Zelalem Nigussie Azene

**Affiliations:** 1 Department of Midwifery, College of Medicine and Health Sciences, Wollo University, Dessie, Ethiopia; 2 Department of Women’s and family health, School of Midwifery, College of Medicine and Health Sciences, University of Gondar, Gondar, Ethiopia

**Keywords:** Antenatal care, Initiation, Continuation, Maternal health care services, Utilization, Ethiopia

## Abstract

**Background:**

Maternity continuum of care is the continuity of maternal healthcare services that a woman uses, which includes antenatal care (ANC 4+), skilled birth attendant (SBA), and postnatal care (PNC) within 48 h of delivery. It is one of the essential strategies for reducing maternal and newborn morbidity and mortality. Therefore, this study aimed to assess the prevalence and factors affecting the initiation and *continuation* of maternal health service utilization among women who delivered in the past one year in *Enemay* district, East Gojjam zone, Ethiopia.

**Methods:**

A community-based cross-sectional study was conducted among six hundred twenty-one (621) women who gave birth in the last one year in Enemay district from February 25 to March 10, *2019.* A simple random sampling technique was used to select the study participants. Data were collected by face-to-face interviewer-administered, pretested, and semi-structured questionnaire. Binary logistic regressions (bi-variable and multivariable) were fitted to identify statistically significant variables. Adjusted Odds Ratio (AOR) with 95% Confidence Interval (CI) was used to declare statistically significant variables on the basis of *p*-value < 0.05 in the multivariable binary logistic regression.

**Results:**

In this study, around 61% of women had antenatal care follow-up. Out of those women having ante natal care follow-up, about 77.5% (95% CI 73, 81.7%) had continued to receiving skilled birth delivery service. Age (AOR = 1.7 95% CI: (1.0, 2.88)), marital status (AOR = 1.6, 95% CI: (1.01, 2.76)), women’s educational status (AOR = 2.9, 95% CI: (1.30, 6.72)), autonomy for health care decision-making (AOR = 3.71, 95%CI: (2.36, 6.02)), exposure to media (AOR = 2.8, 95% CI: (1.78, 4.6)), wanted pregnancy (AOR = 3.6 95% CI: (2.2, 5.95)), and parity (AOR = 0.34, 95%CI: (0.16, 0.71)) were statistically significant variables associated with initiation of antenatal care, whereas educational status of women (AOR = 4.65, 95% CI: (1.37, 15.7)), autonomy for health care decision making (AOR = 2.62, 95% CI:(1.0, 6.82)), and had counseled during antenatal care (AOR = 2.88 95% CI: (1.21, 6.83)) were statistically significant variables associated with the continuation of maternal health care services.

**Conclusions:**

This study demonstrated that the initiation and continuity of maternal health care services are low in the study area. Age, marital status, residence, women’s educational status, health care decision-making autonomy, exposure to media, wanted pregnancy, and parity were factors significantly affecting the initiation of antenatal care. Whereas, *women’s* educational status, health care decision-making autonomy, and *counseling* during antenatal care were predictors influencing the continuation of maternal health care services (antenatal care to skilled birth delivery).

## Background

Utilization of maternal *healthcare* service is essential for the improvement of both maternal and child health, reduction of maternal and child mortality*,* but the continuity of maternal health care utilization has been a challenge in Sub-Saharan Africa. Many mothers who attend antenatal care visits fail to use facility delivery [1, 2]. Antenatal care (ANC) is crucial to provide information for identifying and treating existing social and medical conditions, health promotion, and *screening* of risk factors. However, receiving only antenatal care is not enough since *the majority* of the fatal complications occur at the time of labor and delivery. *Therefore, women* should have *a skilled birth attendant* (SBA) from trained and qualified professionals for safe childbirth as failure to access skilled assistance during delivery results in too many complications [2–4]. Maternal mortality is unacceptably high. About 295,000 women died during and following pregnancy, and childbirth in 2017. The vast majority of these deaths (94%) occurred in low-resource settings and most of the deaths could have been prevented. Most of the deaths occur during labor, delivery and the immediate postpartum period [5]. Ethiopia is one of the countries with the highest maternal mortality levels in the world, with an estimated 412 deaths per 100,000 live births [6].

The maternal mortality ratio and lifetime risk of maternal mortality were 20 and 27 times higher in developing countries when compared to developed ones. For subsequent reduction of maternal mortality, a sustainable development goal was developed, which targets decreasing the global maternal mortality ratio to less than 70 per thousand live births and neonatal mortality to less than 12 per 1000 in all countries by 2030 [7]. In order to achieve this ambitious goal, better access to maternal and reproductive health care services including antenatal care in pregnancy, skilled care during childbirth, and postnatal care service is required [8]. From a global perspective, it is estimated that approximately 80% of maternal deaths and up to two-thirds of neonatal deaths could be prevented if effective health measures are taken during birth and the first week of life [9]. However, utilization of these services in most developing countries is low due to various cultural, socioeconomic, and demographic factors.

Maternal mortality has a negative impact on living children, families, and economic growth. Newborns and children whose mothers died from maternal causes face nutrition deficits, and are less likely to access the needed health care services. Older children drop out of school to care for younger siblings and often choose migration in search of better opportunities and family fragmentation is common following *a maternal death* [11*,* 12]. Maternal mortality *has also impacted* economic growth. A study done in the African region showed that maternal mortality has a statistically significant negative effect on *GDP, which* results in *an annual loss* of US$49,224 [10, 11].

In Ethiopia, 62% of women received antenatal care whereas only 28% were assisted by skilled delivery attendants, which shows a great discontinuity rate [6]. However, studies on factors influencing maternal care initiation or continuation were not available in Ethiopia. The objective of this study was, therefore, to assess the prevalence and factors affecting initiation and continuation of maternal health service utilization among women who delivered in the past one year in Enemy district, East Gojjam zone, Ethiopia.

## Methods

### Study area 

The study was conducted in Enemay district, Amhara regional state, Northwest Ethiopia. The administrative center of this district is Bichena. The district is located in East Gojjam Zone, Amhara regional state, and is 270 kms far from Addis Ababa, the capital of Ethiopia in the Northwest direction and, about 295 km from Bahir Dar city, the capital of Amhara regional state. According to the 2017 statistical figure of Enemay district health office report, the total population in the district is around 198,241. The district has now 7 health centers, 35 health posts, and 1 hospital. All these health institutions are currently providing maternal and child health care services.

### Study design and period

A community-based cross-sectional study was conducted from February 25 to March 10, 2019.

### Population

#### Source population

All women who gave birth in the last one year in Enemay district.

#### Study population

All women who gave birth in the last one year in the selected Kebeles of Enemay district.

**Eligibility Criteria**

**Inclusion criteria**

All women who gave birth in the last one year and lived at least six months in Enemay district.

**Exclusion criteria**

Those women who were severely ill and unable to communicate effectively were excluded from the study.

### Sample size determination and sampling procedures

The sample size was determined for the first and second objectives. Finally, the largest sample size was taken. Single population proportion [12] was used to calculate the sample size for the first objective by assuming the proportion of antenatal care utilization in rural areas of India**,** which was 74% [13] with a 95% confidence level, and 5% marginal error. Based on these assumptions, the total sample size was calculated using the following formula:

$$ \frac{\mathbf{n}=\left(\mathbf{z}\boldsymbol{\upalpha } /\mathbf{2}\right)\mathbf{2}\ \mathbf{p}\ \left(\mathbf{1}-\mathbf{p}\right)}{\mathbf{d2}}\kern0.5em \frac{\mathbf{n}=\left(\mathbf{1}.\mathbf{96}\right)\mathbf{2}\Big(\mathbf{0.74}\left(\mathbf{1}-\mathbf{0.74}\right)}{\left(\mathbf{0.05}\right)\mathbf{2}}=296 $$

Where n = required sample size, Z = critical value for normal distribution at 95% confidence level (1.96), P = Proportion of antenatal care utilization, d = 0.05(5% margin of error). By considering a 10% non-response rate and a design effect of 2, the final sample size was found to be 651.

First, the population was stratified by residence as urban and rural Kebeles. The residential stratification gave 28 rural and 8 urban kebeles**.** Out of those, three urban and eight rural kebeles were selected randomly by using a lottery method. Totally, 11 kebeles were selected from the total.

thirty six kebeles by a simple random sampling method. The number of women who delivered in each selected kebele was taken from the health extension workers’ registration book. Then, the sample was proportionally allocated for each selected kebele. Finally, a simple random sampling technique was employed to select the women by using guidance.

Either of the mothers was interviewed in households having two mothers who gave birth in the last one year in the district. Households that were closed during the data collection period were revisited.

### Data collection tool and procedures

Data were collected using a pretested, semi-structured, and interviewer-administered questionnaire. The questionnaire was developed after reviewing relevant literature. It was prepared originally in English and translated into the local language (Amharic) for the purpose of data collection and then it was translated back to English to maintain the consistency of the tool.

The questionnaire had socio-demographic variables, husband/partner-related variables, and reproductive/obstetrics -related variables. For data collection, 11 diploma nurses and 11 guidance counselors were involved under the supervision of the investigators and supervisors.

### Data quality control

A one-day training was given for data collectors and supervisors. The data were daily checked for completeness and accuracy by the principal investigator and supervisors.

A pretest was conducted on 5% of the sample size in the non-selected kebele of the district to ensure the validity, reliability, and clarity of the data collection instrument**.** Based on the findings from the pretest, modification on the questionnaire was done, and the arrangement of questions was revised.

### Study variables and measurements

**Initiation of maternal health service (utilization of ANC)** was the first outcome variable. It was considered if a woman received one or more ANC visits during her pregnancy period [14] .

**Continuity of maternal health service** was the second outcome variable. Continuity of maternal health service was considered if a woman received at least one antenatal care visit during her pregnancy and gave birth at a health facility [14].

### Statistical analysis

The data were first checked manually for completeness and then coded, cleaned, and entered into Epi data version 3.1 software. The data were exported to Statistical Package for Social Science (SPSS) version 20 for data analysis. Descriptive statistics (like mean, standard deviation, frequencies, and percentages) were used to describe the study population in relation to dependent and independent variables. Results were presented in text and tables.

Binary logistic regression (bivariable and multivariable logistic regression) was used to identify statistically significant independent variables, and independent variables having a *p*-value less than 0.2 in the bi-variable analysis were entered into multivariable binary logistic regression. A p-value< 0.05 in the multivariable analysis was used to declare statistically significant variables. Hosmer-Lemeshow goodness-of-fit test was used to test model fitness and declared good fitted at a p-value of > 0.05. An AOR with 95% CI was reported to show the strength of associations among the independent and outcome variables.

## Results

### Respondents’ socio-demographic characteristics

Of the overall sample required (*N* = 651), 621 participants were included in the study, giving a response rate of 95.4%. The mean age of the women was 30.85 (SD ±6.56) years. The majority 352(56.7%) of the respondents were between 25 and 35 years old, followed by the age group of > 35, years, 159(25.6%). Two hundred twenty-seven (36.6%) were housewives and 225(36.2%) were farmers by occupation. The largest portion of the participants (473 (76.2%)) were Orthodox Christians by religion. Five hundred thirteen (82.6) were currently married and 455(73.3%) were rural residents. Around 46.1% of the women had access to mass media and 77.1% of the women had autonomy in healthcare decision-making (Table 1**).**

**Table 1:** Background characteristics of women who delivered in the past one year in Enemay district, East Gojjam, Ethiopia

Variables	Category	Frequency	Percentage
Age of the respondents	15–24	110	17.7
	25–35	352	56.7
	> 35	159	25.6
Religion	Orthodox	473	76.2
	Others	148	23.8
Marital status of the woman	Single/widowed divorced	108	17.4
	Married	513	82.6
	Cannot read and write	219	35.3
Educational status of the woman	Read and write	125	20.1
	Primary	163	26.2
	Secondary and above	114	18.4
	Housewife	228	36.7
Respondent’s occupation	Merchant	127	20.5
	Farmer	226	36.4
	Employer	40	6.4
Number of children	1–2	298	48.0
	3–4	237	38.2
	> 5	86	13.8
Residence	Rural	455	73.3
	Urban	166	26.7
Autonomy to health care decision making	Yes	479	77.1
	No	142	22.9
Respondent exposure to media	Yes	286	46.1
	No	335	53.9
Wanted pregnancy	Yes	493	79.4
	No	128	20.6
Educational status of husband/partner	cannot read write	165	26.6
	Read and write	172	27.7
	Primary	117	18.8
	Secondary and above	167	26.9
Husband’s occupation	Merchant	213	34.3
	Farmer	353	56.8
	Employer	55	8.9

### Initiation (utilization) of antenatal care

In this study, the *initiation* of maternal health service (women who received at least one antenatal care service) was 60.9% (95% CI: 57.2, 65.1%)**.**

### Factors associated with utilization of antenatal care

Age, marital status, women’s educational status, autonomy for health care decision-making, exposure to media, wanted pregnancy, and parity had significant association with the initiation of antenatal care.

Younger women were more likely to utilize antenatal care (AOR = 1.7 95% CI: (1.0, 2.88)) compared to those older women. Married women had higher odds of utilizing antenatal care (AOR = 1.6, 95% CI: (1.01, 2.76)) compared to those single ones. Women with formal education were more likely to utilize antenatal care (AOR = 2.9, 95% CI: (1.30, 6.72)) compared to those women who had no formal education, and women who had the autonomy in health care decision -making were more likely to utilize antenatal care (AOR = 3.71, 95%CI: (2.36, 6.02)) compared to their counterparts. Women who had exposure to media were more likely to use antenatal care (AOR = 2.8, 95% CI: (1.78, 4.6)) compared to those women who had no exposure to media. Women with wanted pregnancies were more likely to use antenatal care (AOR = 3.6 95% CI: (2.2, 5.95) compared to those women whose pregnancies were unwanted. Para 1–2 women were less likely to utilize antenatal care (AOR = 0.34, 95%CI: (0.16, 0.71) compared to para five and above women. In addition, para 3–4 women were less likely to utilize antenatal care than para five and above women **(**Table 2**).**

**Table 2:** factors associated with utilization of antenatal care among women who delivered in the past one year in Enemay district, East Gojjam, Ethiopia

Variables	Category	ANC		COR 95%CI	AOR 95%CI
		YES	NO		
Age group	15–24	74 (67.3%)	36 (32.7%)	2.030(1.22–3.36)	1.8 (0..88, 3.99)
	25–35	224(63.6%)	128(36.4%)	1.728(1.18–2.52)	1.7 (1.0, 2.88)^*^
	> 35	80 (50.3%)	79 (49.7%)		1
Respondent’s marital status	Married	320 (62.4%)	193(37.6%)	1.43(0.94–2.17)	1.6 (1.01,2.76)*
	Single	58(53.7%)	50(46.3%)	1	1
Educational status of the woman	Have formal education	275(54.2%)	232(45.8%)	7.9(4.1,15.0)	2.9 (1.30, 6.72)*
	Have no formal education	103(90.4%)	11 (9.6%)	1	1
Parity	1–2	196(65.8%)	102(34.2%)	1.52(0.93–2.45)	0.34 (0.16, 0.71)*
	3–4	134(56.5%)	103(43.5%)	1.03(0.63–1.69)	0.46 (0.24, 0.88)*
	> 5	48(55.8%)	38(44.2%)		1
Residence	Rural	249(54.7%)	206(45.3%)	0.35(0.23–0.52)	1.2(0.64, 2.24)
	Urban	129 (77.7%)	37(22.3%)	1	1
Autonomy in health care decision making	Yes	338(70.6%)	141(29.4%)	6.11(4.0–9.23)	3.7(2.36, 6.02)*
	No	40(28.2%)	102(71.8%		1
Media exposure of the woman	Yes	230 (80.4%)	56 (19.6%)	5.18(3.61–7.46)	2.8(1.78, 4.6)*
	No	148 (44.2%)	187(55.8%)	1	1
Wanted pregnancy	Yes	336(68.2%)	157(31.8%)	4.38(2.89–6.63)	3.6(2.2, 5.95)*
	No	42(32.8%)	86(67.2%)	1	1
Husband’s educational status	Have formal education	241(53.1%)	213 (46.9%)	4(2.6,6.24)	1.1(0.60, 2.04)
	Have no formal education	137 (82.0%)	30 (18.0%)	1	1
Husband’s occupational status	Merchant	153 (71.8%)	60 (28.2%)	0.09(0.02–0.40)	0.12(0.02, 0.56)
	Farmer	172(48.7%)	181(51.3%)	0.03(0.09–0.15)	0.1(0.02, 0.55)
	Employer	53(96.4%)	2 (3.6%)	1	1

### Continuity of maternal health care service

In our study, around 61% of the women had antenatal care. Of those women who had antenatal care visit, 77.5% (95% CI: 73, 81.7%) continued to use services provided by skill birth attendants while delivery. On the other hand, 22.5% discontinued utilizing services by skilled birth attendants during delivery**.**

### Factors associated with continuation of maternal health care services

In the multivariable analysis, only *women’s* educational status, autonomy in health care decision -making, and *counseling* during antenatal care were associated with the continuation of maternal health care service (antenatal care to skilled birth delivery).

Women with formal education were more likely to continue from antenatal care to skilled birth delivery (AOR = 4.65, 95% CI: (1.37, 15.7)) compared to those women who had no formal education. Besides, women who had health care decision-making autonomy were 2.6 times more likely to continue from antenatal care to skilled birth delivery (AOR = 2.62, 95% CI:(1.0, 6.82) compared to those who had no autonomy to health care decision-making. Those women who had got counseling services during their pregnancy had higher odds of continuation from antenatal care to skilled birth delivery AOR = 2.88 95% CI: (1.21, 6.83) compared to their counterparts **(**Table 3**).**

**Table 3:** Factors associated with continuation of maternal health care from antenatal care to skill birth delivery among women who delivered in the past one year in Enemay district, East Gojjam

Variables	Category	Continuity of maternal health service		COR 95%CI	AOR
		YES	NO		
Age group	15–24	61 (82.4%)	13 (17.6%)	1.2 (0.56, 2.82)	0.93 (0.28,3.07)
	25–35	169(75.4%)	55 (24.6%)	0.82(0.44, 1.53	0.81(0.34, 1.91)
	> 35	63(78.8%)	17(21.2%)	1	1
Marital Status of the woman	Married	250(78.1%)	70 (21.9%)	1.24(0.65, 2.37)	1.16(0.53,2.56)
	Single/divorced/	43(74.1%)	15 (25.9%)	1	1
Educational status of the woman	Have formal education	97 (94.2%)	6 (5.8%)	6.51(2.74,15.47)	4.65(1.37,15.7)*
	Have no formal education	196 (71.3%)	79(28.7%)	1	
Respondent’s occupation	Housewife	135 (92.5%)	11 (7.5%)	0.64(0.13, 3.04)	3.56(0.47, 26.7)
	Merchant	74 (79.6%)	19(20.4%)	0.2(0.04, 0.92)	0.58(0.09, 3.52)
	Farmer	46 (46.5%)	53(53.5%)	0.04(0.01, 0.20)	0.18(0.02,1.3)
	Employer	38(95%)	2 (5%)	1	1
Parity	1–2	153(78.1%)	43 (21.9%)	0.93(0.43,2.03)	0.65(0.20,2.10)
	3–4	102(76.1%)	32 (23.9%)	0.83(0.37,1.87)	1.13(0.40, 3.15)
	> 5	38 (79.2%)	10 (20.8%)	1	1
Residence	Rural	169(67.9%)	80 (32.1%)	0.08(0.03,0.21)	–
	Urban	124(96.1%)	5(3.9%)	1	–
Autonomy in decision making	Yes	268(79.3%)	70 (20.7%)	2.29(1.15,4.58)	2.62(1.0, 6.82)*
	No	25 (62.5%)	15 (37.5%)	1	1
Media exposure of the respondent	Yes	193(83.9%)	37 (16.1%)	2.5(1.53, 4.09)	1.43(0.74,2.76)
	No	100(67.6%)	48 (32.4%)	1	1
Wanted pregnancy	Yes	261(77.7%)	75 (22.3%)	1.08 (0.51,2.31)	0.65(0.26, 1.65)
	No	32 (76.2%)	10 (23.8%)	1	1
Husband’s educational status	Have formal education	120 (87.6%)	17(12.4%)	2.77(1.55, 4.95)	0.86(0.37, 2.01)
	Have no formal education	173 (71.8%)	68(28.2%)	1	1
Counseling during ANC	Yes	266(79.6%)	68(20.4%)	2.46(1.27,4.77)	2.88(1.21,6.83)*
	No	27 (61.4%)	17(38.6%)	1	

## Discussion

This study examined the factors influencing the initiation and continuation of maternal health care services utilization. Accordingly, the findings of this study indicated that the utilization of antenatal care services was 60.9% (95% CI: 57.2, 65.1%), which is lower than results of studies done in an urban area of Bhubaneswar, India (86.1%) [15], Entebbe, Uganda (96%) [16], Southern Ethiopia (77.4%) [17], Ari Woreda, South Omo Zone (87%) [18] but higher than studies conducted in Menit-Shasha District, Ethiopia (15.5%) [19] and Bangladesh (55%) [20]. This observed variation might be due to differences in socio-demographic characteristics like residence as our study was conducted in both urban and rural areas, differences in the study population, and the measurement tools used across the studies.

Around 77.5% (95% CI: 73, 81.7%) of the women had continued to receive skilled birth delivery once they received antenatal care. This result is consistent with a study done in Cambodia (71%) [21], but higher than studies carried out in nine countries of South Asia and Sub Saharan Africa, which were (28.5%) [2], Nigeria (61.9%) [22], Nepal (39%) [23]. This discrepancy might be due to differences in accessibility of services, measurement, and difference in socio-demographic characteristics of respondents.

*With regard to the associated factors, a significant association was observed with women’s age. It was indicated that being in the age class of* 15–24 *years increases the odds of ANC initiation by* 1.7 times compared to those who were in the age group of 25–35 years. *Similar earlier results were also reported in Addis Ababa* [21–23]*. This may be due to the relative childbearing inexperience (low parity) as they may be newlyweds or adolescents and therefore be more likely to seek out ANC earlier than their older counterparts due to ignorance/limited knowledge of pregnancy. Confounding effect of by parity on age may also have affected the relationship between age and ANC use as low parity was associated with early ANC booking and increased number of ANC contacts* [24, 25]*.*

*Unsurprisingly, marital status was found to be an important predictor in affecting the initiation of antenatal care. It revealed that married women have been found to utilize antenatal care than unmarried ones. This result is congruent with a study conducted earlier* [26]*. This is partially secondary to support from partners and the social acceptability of pregnancy. This is thought to* encourage the attendance of antenatal care. Adolescents and unmarried younger women hide their pregnancy to avoid social embarrassment which in turn delays their initiation of antenatal care visits [27]. In a study done in Ethiopia, single or divorced mothers were more likely to attend antenatal care than mothers who were married [28]. Furthermore, the findings of this study revealed that formal education was associated with the initiation of antenatal care. This result concurs with former studies [29, 30]. It was reported that improving women’s education increases the utilization of maternal healthcare services, including antenatal care. In a study conducted in Sudan, it was revealed that lack of maternal education increased the odds of non-use of antenatal care [31]. This could be due to the fact that women with formal education have greater confidence to take actions regarding their own health and they have awareness on advantage of utilizing health services compared to women who had no education [32, 33]. In addition to that, education makes women more empowered and confident by giving them information on their health, so they can decide to seek care during pregnancy or delivery [34].

Moreover, in our analysis, women’s health care decision-making autonomy was positively associated with the initiation of antenatal care. This finding is consistent with the studies conducted so far [35, 36]. The plausible explanation might be that autonomous women would make the right choices and access quality healthcare at all times particularly during antenatal care [37].

This study also revealed that women who have been using mass media were 2.8 times more likely to utilize antenatal care services than women who did not use any type of mass media. Similar findings were reported in the study done in Ethiopia in 2017, [38], 2019 [39], and Indonesia [40]. This might be because using mass media may increase the knowledge and practices of the women to utilize proper antenatal care for the benefits of the health of the mother and newborn babies.

Our analysis showed that women whose pregnancies were wanted were more likely to receive antenatal care. This is supported by studies conducted in other countries [41–43]. It is possible that women whose pregnancies were unintended may fear the social ramifications of an unplanned pregnancy, so they may avoid health services utilization.

Interestingly, this study indicated that an increase in parity increases the likelihood of uptake/ initiation of antenatal care. There is no study in agreement with this finding. The possible explanation for this result might be that when parity increases women might have past obstetrics complications which lead them to seek maternal health care services including antenatal care.

Counseling during antenatal care was significantly associated with the continuation of antenatal care to skilled delivery. The possible reason might be that women who were counseled during antenatal care might receive information about birth preparedness and complication readiness including being assisted by skilled birth attendants during delivery. Educational status was also another factor affecting both the initiation and continuation of maternal health care service utilization. This is in line with studies done in Pakistan and Cambodia [44, 45]. The possible justification for this might be that those educated women might have better health knowledge about the importance of delivery with skilled birth attendants. The other possible reason could be that education is likely to enhance and develop greater confidence and capability to make decisions about their own health, and educated women may probably have a good chance to approach the written information about maternal health service.

Having the autonomy in health care decision-making was also positively associated with both initiation and continuation of maternal health care service utilization which is consistent with a study conducted in Pakistan [44]. A possible explanation could be that women who have autonomy in decision-making are more likely to have a higher level of autonomy in health care, which might lessen their reproductive behavior risks [46]. Another study has confirmed that women’s control over household resources (ability to keep money aside) has a significant positive effect on both the demand for prenatal care and the probability of hospital delivery [47].

### Limitations of the study

This study acknowledged some important possible limitations that should be considered when interpreting the results. First, the study was cross-sectional, a design that does not permit to establish cause-effect relationships. Second, social desirability bias and recall bias might be introduced as information in the survey was based on self-reports of the study participants.

### The added value of this work

This study tried to assess the magnitude of the initiation of antenatal care service and the continuation of antenatal care to institutional delivery. Moreover, the determinant factors of initiation and continuation of the services have been explored, which other earlier studies did not address.

### Implications for policymakers

The evidence from these findings calls for policymakers to play a role in enhancing the utilization of antenatal care and skilled birth attendant (institutional delivery) services through scaling up the awareness of those illiterate women at the community level about the service through appropriate information outlets such as mainstream media, proper counseling during antenatal care follow-up.

## Conclusions

This study showed that the initiation and continuity of maternal health care services utilization are low in the study area compared to the national target. Age, marital status, residence, women’s educational status, autonomy to health care decision making, exposure to media, wanted pregnancy, and parity were factors significantly affecting the initiation of antenatal care, whereas *women’s* educational status, autonomy to health care decision making, and *counseling* during antenatal care were predictors influencing the continuation of maternal health care services (antenatal care to skilled birth delivery).

## Data Availability

The dataset analyzed during the current study available from the corresponding author on reasonable request.

## Abbreviations

ANC: Antenatal Care
SBA: Skilled Birth Attendant
AOR: Adjusted Odds Ratio
CI: Confidence Interval
CSA: Central Statistics Agency
SPSS: Statistical Package for Social Science
WHO: World Health Organization
GDP: Gross domestic product
